# Anaerobic Digestion of Chicken Manure in the Presence of Magnetite, Granular Activated Carbon, and Biochar: Operation of Anaerobic Reactors and Microbial Community Structure

**DOI:** 10.3390/microorganisms10071422

**Published:** 2022-07-14

**Authors:** Elvira E. Ziganshina, Ayrat M. Ziganshin

**Affiliations:** Department of Microbiology, Institute of Fundamental Medicine and Biology, Kazan (Volga Region) Federal University, 420008 Kazan, Russia; elvira.ziganshina@kpfu.ru

**Keywords:** anaerobic digestion, methane, chicken manure, magnetite, granular activated carbon, biochar, anaerobic bacteria, methanogenic archaea

## Abstract

The influence of magnetite nanoparticles, granular activated carbon (GAC), and biochar, as well as their combinations on the anaerobic digestion of chicken manure and the structure of microbial communities was studied. The addition of magnetite, GAC, and biochar increased the rate of methane production and the total methane yield. It has been observed that these additives stimulated anaerobic microorganisms to reduce the concentration of accumulated volatile organic acids. Various bacterial species within the classes *Bacteroidia* and *Clostridia* were found at higher levels in the anaerobic reactors but in different proportions depending on the experiment. Members of the genera *Methanosarcina*, *Methanobacterium*, *Methanothrix*, and *Methanoculleus* were mainly detected within the archaeal communities in the anaerobic reactors. Compared to the 16S rRNA gene-based study, the *mcrA* gene approach allowed a higher level of *Methanosarcina* in the system with GAC + magnetite to be detected. Based on our findings, the combined use of granular activated carbon and magnetite at appropriate dosages will improve biomethane production.

## 1. Introduction

Anaerobic digestion is an attractive and widely used technology to maximize the metabolic ability of microorganisms to convert the organic fraction of various organic wastes into biogas. Enclosed biogas reactors can prevent potential greenhouse gas emissions and odors. A significant amount of manure is formed annually in the world, the uncontrolled decomposition of which can lead to a deterioration in the quality of the atmosphere, pollution of soil, and contamination of water resources. However, manure as a suitable substrate for the anaerobic digestion process has already received a large amount of attention due to its high moisture content, availability of organic matter, and other various important compounds. Anaerobic digestion is clearly a more appropriate technology for the treatment of animal waste, as it converts waste into bioenergy while addressing the manure pollution problem and energy scarcity [1,2,3]. Thus, biomethane released from anaerobic reactors can be further used for the local production of heat and electricity [4]. In addition, CO_2_ from biogas and nutrients from digestate can be used to produce algal biomass [5,6,7].

As the commercial opportunities for selling chicken meat expand, global chicken farming is on the rise, followed by a significant production of chicken wastes. Treatment of chicken manure through the anaerobic digestion process is becoming increasingly common as an important component in the search for sustainable energy sources. This technology further stabilizes this waste and minimizes its impact on the environment and ecological systems [8,9]. However, anaerobic digestion of such a substrate with a high content of undigested protein and uric acid often leads to process instability due to the accumulation of ammonium and free ammonia. This leads to reduced methane production, fluctuations in pH and alkalinity, and accumulation of organic acids [10,11]. Anaerobic digestion of such substrates also leads to the formation of sulfides, which are not only toxic to various microorganisms, but additionally form complexes with metals, leading to a decrease in the bioavailability of trace elements necessary for the activity of microorganisms [12]. Despite this, many positive factors continue to drive the development of commercial biogas plants to operate on nitrogen-rich substrates, requiring different solutions and new strategies to address the associated challenges.

Anaerobic digestion of biomass is divided into four stages: hydrolysis, acidogenesis, acetogenesis, and methanogenesis. Anaerobic digestion is a complex microbial process in which fermentative bacteria transform complex organic substances into various simple metabolites, including acetic acid, molecular hydrogen, formic acid, and methanol, which are ultimately utilized by methanogenic archaea [13]. However, this syntrophy may be impaired due to the accumulation of inhibitory metabolites. For example, high concentrations of volatile fatty acids (VFAs), including acetic, propionic, and butyric acids, can acidify the content of anaerobic reactors, leading to inhibition of the activity of the methanogenic communities [14,15]. In recent years, it has been demonstrated that an effective approach to enhance the methanogenic transformation of various VFAs is the process with the addition of conductive materials [16,17,18,19,20,21]. Among the explanations for the optimization of anaerobic biomass conversion using conductive materials, the researchers note the provision of direct interspecies electron transfer (DIET) between bacteria, which are involved in the decomposition of organic matter, and archaea, which directly produce methane. The process in which electrons transfer directly from electron donors to electron acceptors via microbial nanowires or non-biological conductive materials in anaerobic systems (e.g., iron oxides, GAC, and biochar) is called DIET. The introduction of these conductive materials to anaerobic digesters may ultimately stimulate the more efficient degradation of various VFAs into biomethane under acidic conditions [14], high ammonium concentrations [19,21], and different temperature conditions [14,15,16,17,18,19,20,21]. However, little research has been conducted to date on the effects of adding various conductive additives, either alone or together, on the anaerobic digestion of nitrogen-rich chicken manure.

Therefore, in the presented study, the effect of magnetite, GAC, and biochar on the anaerobic conversion of chicken manure was investigated. The 16S rRNA genes of bacteria and archaea as well as *mcrA* genes of methanogenic archaea have also been characterized to describe the structure of microbial communities in these anaerobic systems. The results obtained in this study will contribute to the improvement of the anaerobic digestion of chicken manure in practice.

## 2. Materials and Methods

### 2.1. Substrate, Inoculum, and Additives

Chicken manure with a total solids (TS) content of 64.6 ± 0.47% and a volatile solids (VS) content of 52.2 ± 0.51% used for the anaerobic digestion tests was obtained from a local chicken farm (the Republic of Tatarstan, Russia). The collected chicken manure was then stored at +4 °C. The digestate used as an inoculum in the experiments was obtained from a mesophilic (38 °C) laboratory-scale biogas reactor processing cow manure. The inoculum for the first batch tests had the TS content of 3.65 ± 0.12% and the VS content of 2.56 ± 0.10%. The inoculum for the second batch tests had the TS content of 4.36 ± 0.12% and the VS content of 2.69 ± 0.11%. Magnetite (Fe_3_O_4_) nanopowder (50–100 nm particle size, Sigma–Aldrich, St. Louis, USA), granular activated carbon produced from coconut shell via physical and chemical activation (GAC; 0.5–2.4 mm particle size; Germany), and biochar produced from bark-free wood via pyrolysis (1.0–5.0 mm particle size; Russia) were used in this study.

### 2.2. Anaerobic Digestion Experimental Design

Anaerobic digestion batch assays were performed using AMPTS II Light complete systems (Bioprocess Control, Sweden) at 38 °C in 2000 mL glass vessels with a working volume of 1600 mL for 25–30 days. During the first experiments, the 2000 mL flasks contained 45 g of chicken manure, 1320 g of inoculum, and 235 g of tap water (the final TS content of 5% was achieved). The ratio of inoculum to substrate was 33.79 g/23.47 g (1.44; calculated as VS). In another series of experiments, the concentration of chicken manure was almost doubled. Thus, during the second experiments, the 2000 mL flasks contained 89.5 g of chicken manure, 1240 g of inoculum, and 270.5 g of tap water (the final TS content of 7% was achieved). The ratio of inoculum to substrate was 33.36 g/46.71 g (0.71; calculated as VS). Blank rectors were also used to compensate for the level of CH_4_ generated by the inoculum itself. The control reactors were operated without any additives. Magnetite (50 mg per 1 g of TS), GAC (5.0 g L^−1^), and biochar (5.0 g L^−1^) were added separately to the experimental reactors. The dosage of additives was chosen based on the best performance shown by us recently [15,20], but for other substrates. In addition, magnetite and GAC, as well as magnetite and biochar, were also combined to observe their mutual influence on the anaerobic digestion of chicken manure. All anaerobic digestion experiments were conducted in duplicate. Before the start of the experiments, the anaerobic reactors were purged with N_2_ for 2 min to remove O_2_. All anaerobic reactors were agitated at 60 rpm for 1 min with a 3 min rest interval.

### 2.3. Analytical Methods

Total solids, volatile solids, pH, volatile organic acids (VOA), and total ammonia nitrogen (TAN) were measured following standard methods as explained in detail previously [20,21]. Samples for these analyses were periodically collected, and the analyses were repeated three times. The amount of CH_4_ generated by the AMPTS II Light instruments was adjusted to standard conditions. The methane content in biogas was periodically measured using a Clarus 580 gas chromatograph (Perkin Elmer, Singapore).

### 2.4. Microbial Community Analysis

The microbial communities’ structure in the reactors of the second batch tests on day 6 was characterized by molecular methods targeting 16S rRNA and *mcrA* genes, as explained in detail previously [15,20]. Only one replicate from duplicate treatments was analyzed. Briefly, a FastDNA spin kit for soil (MP Biomedicals, Irvine, CA, USA) was applied to extract the total DNA. Bacterial 16S rRNA gene fragments were amplified with the primers Bakt_341F (5′-CCT ACG GGN GGC WGC AG-3′) and Bakt_805R (5′-GAC TAC HVG GGT ATC TAA TCC-3′). Archaeal 16S rRNA gene fragments were amplified with the primers Arch349F (5′-GYG CAS CAG KCG MGA AW-3′) and Arch806R (5′-GGA CTA CVS GGG TAT CTA AT-3′). Moreover, the primers mlas (5′-GGT GGT GTM GGD TTC ACM CAR TA-3′) and mcrA-rev (5′-CGT TCA TBG CGT AGT TVG GRT AGT-3′) were used to amplify the *mcrA* gene of methanogenic archaea. Amplicon sequencing was conducted using an Illumina MiSeq system with 2 × 300 bp reads. The sequence data were then analyzed as detailed previously [15,20,21,22] and available on request.

### 2.5. Statistical Analysis

Tukey method and 95% confidence were used to compare differences (Minitab software version 20.2.0, State College, PA, USA).

## 3. Results and Discussion

### 3.1. Process Stability and Methane Generation (TS Content of 5%)

According to our preliminary experiments on the changes in total methane production and methane flow rates caused by different concentrations of magnetite, GAC, and biochar (data not shown), these additives were tested at optimal concentrations during the anaerobic conversion of chicken manure, considering the stimulating effect and further practical application. Thus, five different conditions were controlled during the experimental period: control reactors (C1), reactors supplemented with Fe_3_O_4_ (M1), reactors supplemented with GAC (G1), reactors supplemented with GAC and magnetite (GM1), and reactors supplemented with biochar (B1). The biogas reactors were operated for 25 days, and four samples were obtained to analyze the parameters of the process.

**Figure 1:**
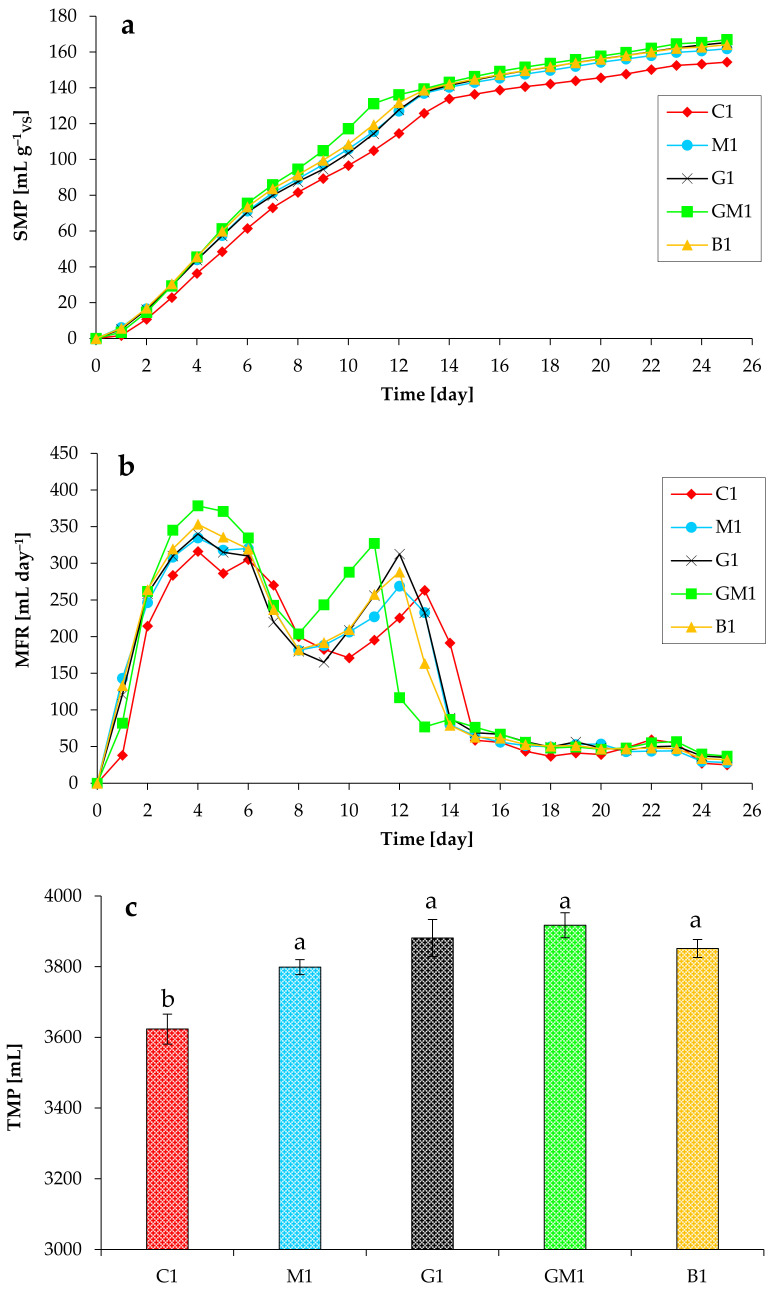
Impact of various additives on specific CH_4_ production (SMP; (**a**)), CH_4_ flow rate (MFR; (**b**)), and total CH_4_ production (TMP; (**c**)) during anaerobic digestion of chicken manure (TS of 5%). Means that do not share a letter are significantly different (**c**).

Figure 1 demonstrates the CH_4_ production from all anaerobic systems. As can be observed, CH_4_ was efficiently produced in all experiments. This indicated that the chicken manure was suitable for the anaerobic digestion process, though these wastes generated lower levels of methane compared with our previous research [21]. After a very short lag phase (several hours), the anaerobic reactors started producing CH_4_ and nearly finished within 25 days. The addition of Fe_3_O_4_, GAC, and biochar increased the CH_4_ production rate. The mean specific methane production (SMP) from the C1, M1, G1, GM1, and B1 reactors achieved 105 mL g^−1^_VS_, 116 mL g^−1^_VS_, 114 mL g^−1^_VS_, 131 mL g^−1^_VS_, and 119 mL g^−1^_VS_ on day 11, correspondingly (Figure 1a). Significant differences (at α = 0.05) in final SMP values were observed in the absence and presence of different additives (except for M1).

The average maximum peaks of CH_4_ production in all five experiments during the first week of experiments were 316 mL (C1), 335 mL (M1), 340 mL (G1), 379 mL (GM1), and 353 mL (B1), respectively, and the corresponding time was day 4 (Figure 1b). The average maximum peaks of CH_4_ production in all five experiments during the second week of experiments were 263 mL (C1), 269 mL (M1), 313 mL (G1), 327 mL (GM1), and 288 mL (B1), and the corresponding time was day 13 (for C1), day 12 (for M1, G1, and B1), and day 11 (for GM1). In the group with GAC + magnetite, most of CH_4_ was generated by day 12, whereas in the control group, most of CH_4_ was produced only by day 15. These results indicate that the addition of magnetite, GAC, and biochar to the anaerobic reactors results in an increase in the rate of CH_4_ production, with GAC and magnetite, when used jointly, being the most active compounds. Thus, the maximum methane production rate from the GM1 reactors significantly increased (at α = 0.05) by 20% compared to the C1 reactors. Finally, these supplements also improved the ultimate production of methane (Figure 1c).

**Figure 2:**
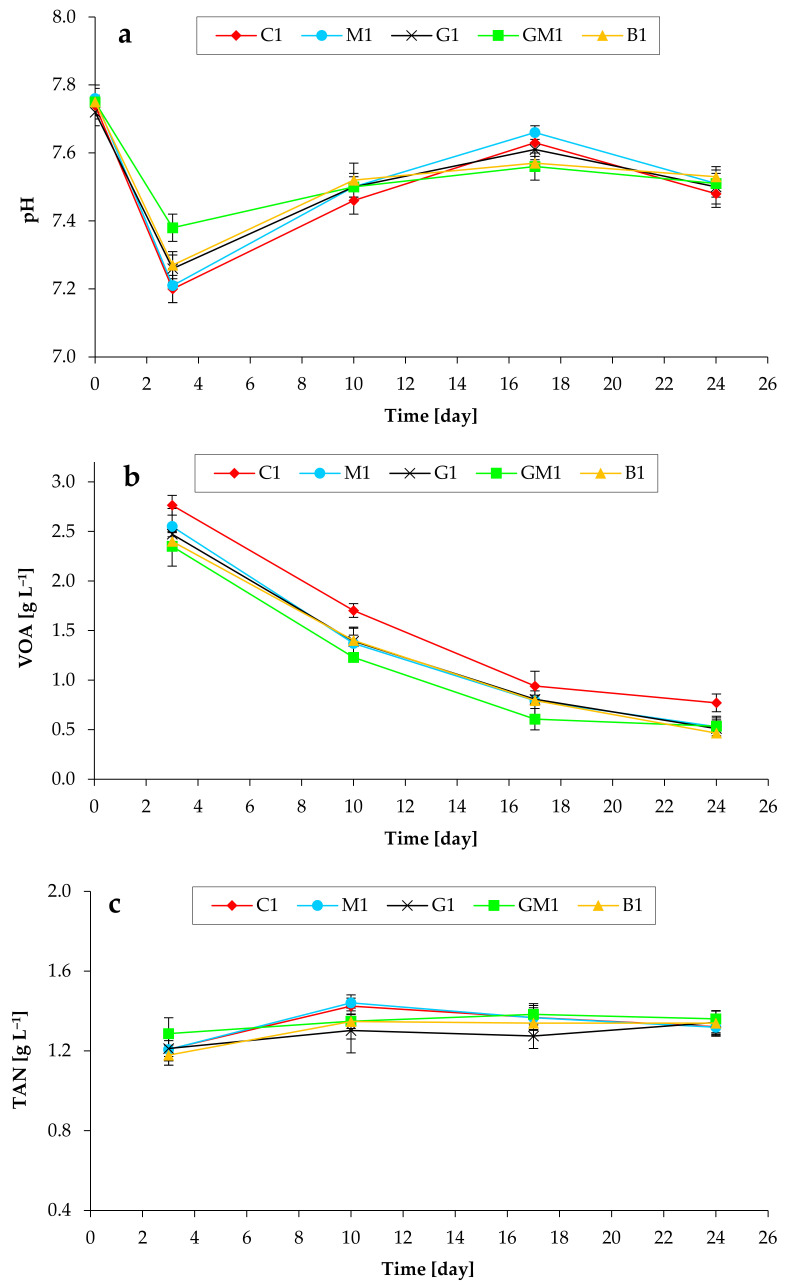
Impact of various additives addition on pH changes (**a**), volatile organic acids (VOA) concentrations (**b**), and total ammonia nitrogen (TAN) concentrations (**c**) during anaerobic digestion of chicken manure (TS of 5%).

Figure 2 shows the pH, VOA, and TAN values in biogas reactors. Throughout the process, the mean pH values in the anaerobic digesters initially decreased from ~7.7 to 7.2–7.4 (on day 3), but finally achieved the values of ~7.5, as shown in Figure 2a. The lowest pH values were observed in C1 on day 3. In addition, temporal changes in volatile organic acids (VOA) concentrations were observed (Figure 2b). It has been observed that VOA consumption by microorganisms is clearly dependent on the addition of various supplements. The addition of magnetite, GAC, and biochar reduced the cumulative VOA concentrations during the anaerobic digestion period, with the GAC/magnetite being more active compounds. Thus, the mean acid capacity in the anaerobic digesters with additives was in the range of 2.4–2.6 g L^−1^, whereas in control experiments it was 2.8 g L^−1^ (on day 3). This indicates that the CH_4_-forming activity in the control batch tests was lower at the beginning of the treatments. Finally, most of the organic acids in all batch tests were efficiently converted to biomethane. It should be additionally noted that some organic acids were derived from the inoculum.

Our results are in line with the results of a number of other research works that have indicated the effectiveness of the utilization of organic acids by microbes in the presence of additives such as magnetite [15,17], GAC [16,18,21], and biochar [23,24], but in other biogas-producing anaerobic systems.

Anaerobic digestion of chicken manure was accompanied by the accumulation of TAN (Figure 2c). Comparable TAN values were detected in all reactors (1.3–1.4 g L^−1^ on day 25). The toxicity of ammonium should not substantially impact the methanogenic communities’ activity in our systems because the concentrations of NH_4_^+^-N in all batch tests were comparable, and these concentrations are usually observed in well-performed biogas generating reactors [25].

### 3.2. Process Stability and Methane Generation (TS Content of 7%)

In this series of experiments, the TS content in the reactors was increased to 7%. Compared with the low solids anaerobic digestion process, high solids anaerobic digestion is more feasible due to the relatively higher loading rate, higher methane yield, smaller footprint, and lower energy consumption. Six different conditions were monitored during the entire period: control reactors (C2), reactors supplemented with magnetite (M2), reactors supplemented with GAC (G2), reactors supplemented with GAC and magnetite (GM2), reactors supplemented with biochar (B2), and reactors supplemented with biochar and magnetite (BM2). The mesophilic reactors were operated for 30 days, and four samples were obtained from each batch test to analyze the process parameters.

**Figure 3:**
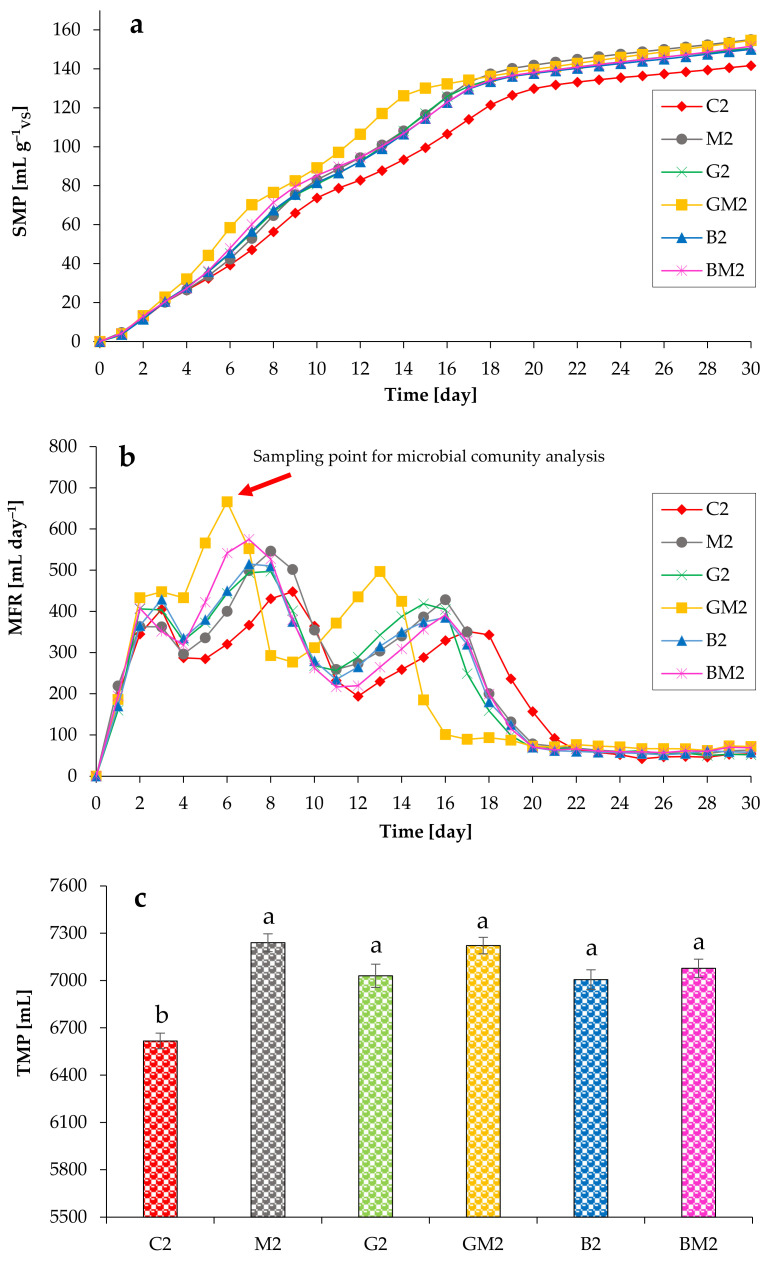
Impact of various additives on SMP (**a**), MFR (**b**), and TMP (**c**) during anaerobic digestion of chicken manure (TS of 7%). Means that do not share a letter are significantly different (**c**).

Figure 3 illustrates the CH_4_ yield from the chicken manure-containing anaerobic systems in the absence/presence of various additives. After a few hours, the anaerobic digesters began to produce CH_4_ and almost finished their work in 30 days. The addition of Fe_3_O_4_, GAC, and biochar, as well as their combinations, increased the CH_4_ production rate. After two days of anaerobic digestion, the GAC/magnetite-containing reactors generated more CH_4_ than other systems. Thus, the average SMP from the reactors C2, M2, G2, GM2, B2, and BM2 reached 93 mL g^−1^_VS_, 108 mL g^−1^_VS_, 108 mL g^−1^_VS_, 126 mL g^−1^_VS_, 107 mL g^−1^_VS_, and 107 mL g^−1^_VS_ on day 14, correspondingly (Figure 3a). Significant differences (at α = 0.05) in final SMP values were observed in the absence and presence of different additives.

The maximum two peaks of CH_4_ generation in all six anaerobic systems (C2, M2, G2, GM2, B2, and BM2) were 448/352 mL, 546/428 mL, 497/419 mL, 666/497 mL, 515/385 mL, and 575/392 mL, accordingly, and the corresponding times were the days 9/17 (for C2), days 8/16 (for M2), days 8/15 (for G2), days 6/13 (for GM2), and days 7/16 (for B2, BM2) (Figure 3b). In group with GAC + magnetite, most of CH_4_ was generated by day 15, whereas its production tended to plateau on day 18 and 20 in other additives-containing groups and control group, respectively. These results indicate that the addition of magnetite, GAC, and biochar increases the rate of CH_4_ production, with GAC and magnetite being the most active supplements when used together. Thus, the maximum methane production rate from the GM2 reactors significantly increased (at α = 0.05) by 48% compared to the C2 reactors. Finally, these additives also improved the ultimate production of methane (Figure 3c).

**Figure 4:**
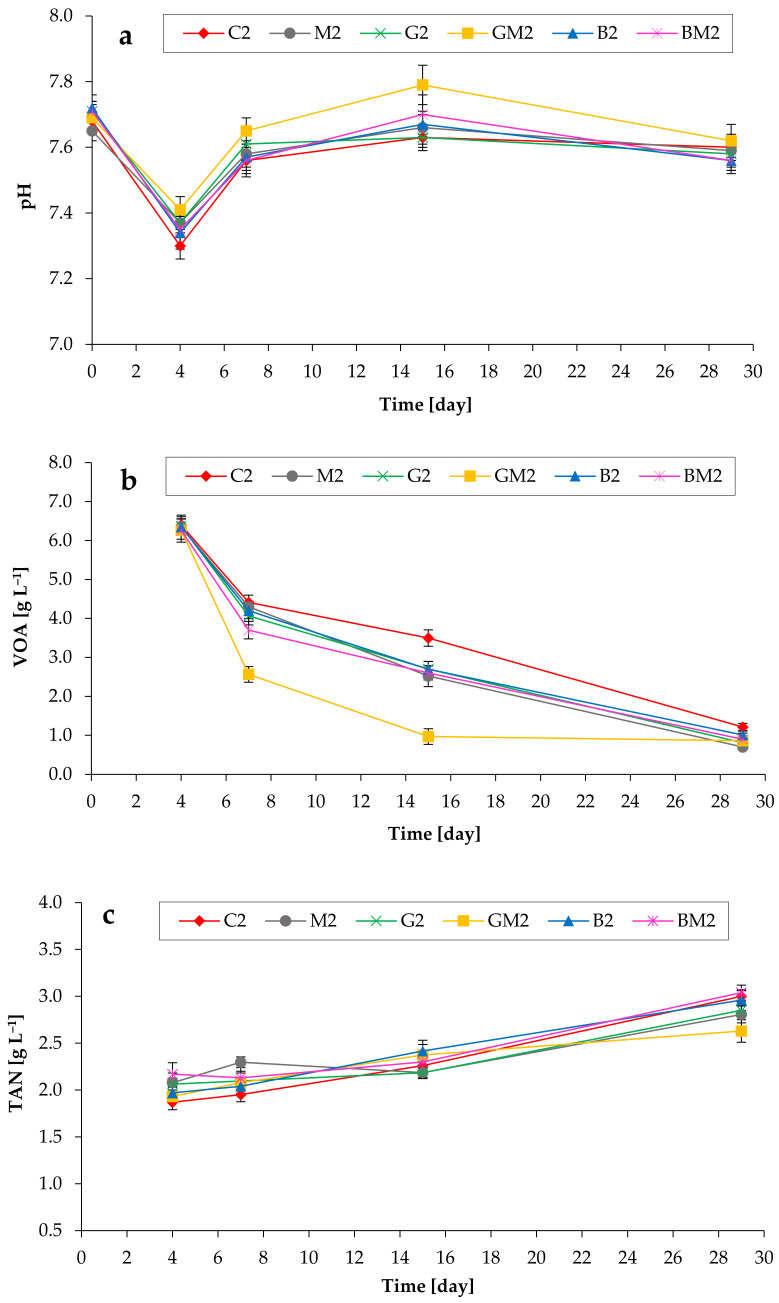
Impact of various additives addition on pH changes (**a**), VOA concentrations (**b**), and TAN concentrations (**c**) during anaerobic digestion of chicken manure (TS of 7%).

During the experiments, the mean pH values in the anaerobic digesters first dropped from ~7.7 to 7.3–7.4 (on day 4), but then achieved the final values of ~7.6 (Figure 4a). In addition, temporal changes in VOA concentrations were observed (Figure 4b). The utilization of VOA by microbes has been influenced by the addition of various additives. The addition of Fe_3_O_4_, GAC, and biochar, as well as their combinations, significantly decreased the concentrations of cumulated VOA during the whole anaerobic digestion period, with the GAC/Fe_3_O_4_ being more active additives. For example, the mean acid capacity in the C2, M2, G2, GM2, B2, and BM2 achieved 4.4 g L^−1^, 4.3 g L^−1^, 4.1 g L^−1^, 2.6 g L^−1^, 4.2 g L^−1^, and 3.7 g L^−1^, respectively (on day 7). This shows that CH_4_-generating microbial activity in the reactors containing no additives was lower at the beginning of the batch tests. Finally, during the anaerobic process, most of the accumulated acids in all reactors at the beginning of the tests were effectively utilized by microorganisms. Anaerobic digestion of chicken manure also resulted in the accumulation of high amounts of TAN (Figure 4c). Higher substrate concentration resulted in higher ammonia levels (final 2.6–3.0 g L^−1^ of TAN depending on the reactor).

Our results are consistent with the positive effects of GAC, biochar, and magnetite on CH_4_ production during the anaerobic digestion of different other substrates as reported in several other works [15,16,17,18,19,20,21,26,27]. Yang et al. [16] indicated that the addition of GAC to biogas reactors (33.3 g L^−1^) enhanced the CH_4_ production by 17.4% over the course of anaerobic sludge digestion. Capson-Tojo et al. [18] showed that activated carbon (10 g L^−1^) in biogas reactors promoted biomass acclimatization. This led to the improvement in the consumption of acetic acid and an increase in the production of CH_4_ from food wastes. Ziganshina et al. [20] demonstrated the effectiveness of the addition of GAC (5–10 g L^−1^) on anaerobic digestion of beet pulp and distillers grains with solubles.

The effects of dairy manure-derived biochar (at concentrations of 1–10 g L^−1^) on anaerobic digestion of dairy manure under psychrophilic, mesophilic, and thermophilic conditions was additionally investigated by Jang et al. [23]. Fagbohungbe et al. [26] studied the effect of wood biochar, coconut shell biochar, and rice husk biochar, as well as biochar ratios, on the anaerobic digestion of citrus peels. Both collectives reported that the addition of biochar to biogas reactors shortened the length of the lag phase and stimulated the CH_4_ production at higher levels. Significant improvements in CH_4_ production have been noted for all types of biochar.

Wang et al. [17] reported that the addition of magnetite nanoparticles (50 mg per 1 g of TS) to biogas reactors decreased the short-chain fatty acids concentration and enhanced the CH_4_ generation during anaerobic digestion of high-solids sewage sludge. Furthermore, Suanon et al. [27] used iron nanoparticles (0.5% and 1%) for the anaerobic digestion of sludge. The authors demonstrated that the addition of Fe_3_O_4_ (at a level of 0.5%) enhanced the production of biogas and improved stabilization of metals in the digestion mixture. Similarly, various iron-bearing minerals also affect the biodegradation of different xenobiotics [28].

The physical characteristics of GAC and biochar stimulate the formation of biofilms on their surfaces. This can increase the resistance of microorganisms to different toxic compounds and, finally, can increase microbial activity [26,29]. Moreover, conductive carbon-containing materials, such as GAC and biochar, as well as magnetite particles, have been shown to promote DIET, accelerating the anaerobic digestion process [30]. Thus, it can be assumed that a better understanding of the mechanisms of DIET will eventually lead to improvements in the design of anaerobic biogas reactors, which will contribute to the DIET mechanism and further improve the anaerobic digestion process. However, this efficacy must be evaluated with the application of more substrates and anaerobic microorganisms to clarify the anaerobic process in more detail.

### 3.3. Microbial Community Structure in Series of Experiments with TS of 7%

In this study, the effect of separate addition of magnetite, coconut-based granular activated carbon, and wood-derived biochar, as well as the effect of the combined addition of GAC and magnetite, biochar and magnetite into anaerobic reactors on bacterial and archaeal communities during anaerobic digestion of chicken manure was additionally investigated (in the exponential phase of methanogenesis). Only one replicate from duplicate treatments was analyzed.

A total of 374,567 high-quality bacterial 16S rRNA gene sequences were generated, with an average of 62,428 reads per sample (from 42,171 to 86,652) by using an Illumina sequencing platform. Operational taxonomic units (OTUs) in samples from the reactor C2 (without any additives) and the experimental reactors M2, G2, GM2, B2, and BM2 were obtained based on relative abundance >0.01%. Alpha diversity indices were calculated on the OTU level to estimate the diversity and richness of the bacterial community in each sample. Data are summarized in Table 1. Lower bacterial diversity in the sample from the reactor without any additives was observed.

The predominant phyla present in samples from the control and experimental reactors were identified as members of the *Bacteroidetes* and *Firmicutes* with a wide range of abilities such as hydrolysis, and fermentation with the production of organic acids and some gases. Members of these groups appear to be important representatives of the bacterial communities in the anaerobic digestion of chicken manure, and this is consistent with other studies [8,31].

**Figure 5:**
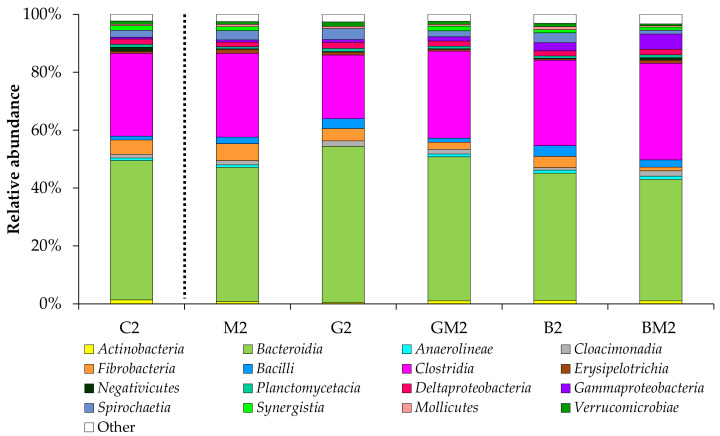
Taxonomic composition of bacterial communities in the anaerobic reactors (sampled on day 6; class level). Classes with abundances below 1% are summarized as “other”.

The relative abundance of bacterial classes in all samples is shown in Figure 5. The most abundant classes in the sample from the control reactor C2 were *Bacteroidia* (48%) and *Clostridia* (29%). These classes also prevailed in all reactors with additives: the relative abundance of *Bacteroidia* reached 42–54%, while the relative abundance of *Clostridia* achieved 22–33%. Other classes included *Fibrobacteria*, *Bacilli*, *Deltaproteobacteria*, *Gammaproteobacteria*, *Spirochaetia*, *Synergistia*, *Verrucomicrobiae*, *Cloacimonadia*, and some others. Like the control reactor, the reactors with supplements showed only small taxonomic shifts on the class level, which can highlight the robustness of the core microbiome.

**Figure 6:**
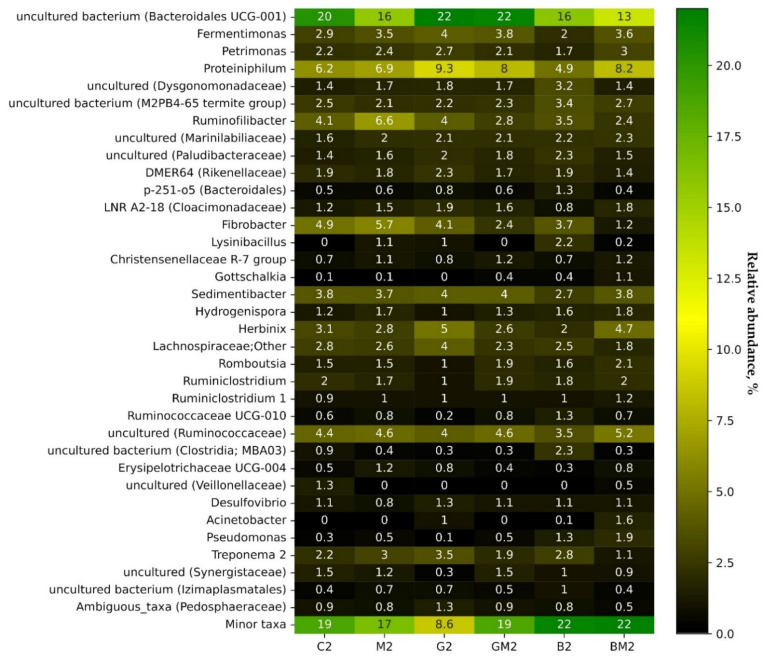
Heatmap demonstrating the relative abundances of bacterial genera in the reactors (sampled on day 6). Only genera comprising at least 1% relative abundance in at least one sample are presented.

To reveal differences in the relative abundance of bacteria and to suggest their possible functions, the taxonomic distribution of bacterial communities was determined on the genus level. Figure 6 demonstrates a heatmap of the relative abundance of the most common bacterial genera detected in the samples, including samples from the control treatment and treatments with magnetite, GAC, biochar, and their combinations.

All reactors were characterized by the predominance of *Bacteroidales* UCG-001 within *Bacteroidia*. The order *Bacteroidales* is known for its involvement in the hydrolysis, acidogenesis, and acetogenesis steps [32,33]. *Fermentimonas*, *Proteiniphilum,* and uncultured members of the family *Dysgonomonadaceae* (*Bacteroidales*) were slightly higher in the reactor with the simultaneous addition of GAC and magnetite (compared to the control reactor) as the most active supplements in our experiments (+31.0%, +29.0%, and +21.4%, respectively). The main fermentation products of members of the genera *Fermentimonas* and *Proteiniphilum* are acetic acid and propionic acid [34]. It should be noted that the relative abundance of these microbes was also higher in the other experimental reactors (with the exception of B2 for the genera *Fermentimonas* and *Proteiniphilum*). Interestingly, an increased abundance of members of *Proteiniphilum* could promote methane production by accelerating the rate of propionate degradation [35] and its potential electroactivity, as it has been described as a member of electroactive consortia [36]. Among other representatives of the bacterial community, the level of which slightly increased in the reactor with the simultaneous addition of GAC and Fe_3_O_4_ (compared to the control reactor), we observed several members of the taxa *Marinilabiliaceae*, *Paludibacteraceae*, LNR A2-18, *Christensenellaceae* R-7 group, *Romboutsia*, and *Ruminococcaceae*. Thus, a comparative taxonomic variability of samples of the experimental reactors on the genus level was noted; however, the introduction of agents did not lead to a sharp change in the taxonomic profile, which may indicate the stability of the composition of bacterial communities. However, the relative abundance of a bacterial group does not reflect its absolute abundance.

To reveal the microorganisms responsible for the final stage of the anaerobic process the taxonomic distribution of archaeal communities and the diversity of methanogens were also determined. A total of 378,147 high-quality archaeal 16S rRNA gene sequences were obtained, and the average number of reads per sample was 63,024 (from 38,430 to 91,651). Alpha diversity indices are presented in Table 1. The number of archaeal OTUs was almost identical in all batch tests.

**Figure 7:**
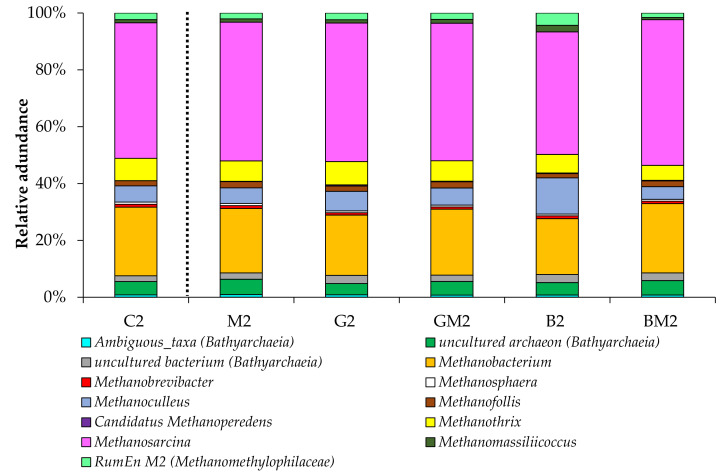
Taxonomic composition of archaeal communities in the anaerobic reactors (sampled on day 6; genus level).

The dominant archaeal classes observed in our experiments were *Methanomicrobia* and *Methanobacteria*. This is consistent with the data of other works reporting the predominance of these groups of methanogens during anaerobic digestion of chicken manure [21,37,38]. Other observed phyla were *Thermoplasmata* within *Euryarchaeota* (ranging from 2 to 7%) and *Bathyarchaeia* within *Crenarchaeota* (in the range of 7–9%). Recent findings indicate that the phylogenetic diversity of methanogens may be much higher, with the inclusion of archaeal phyla outside the *Euryarchaeota* such as *Bathyarchaeia* [39,40], which allows us to note that the archaeal communities in our reactors included a wide variety of methanogens. The archaeal community structure analysis showed that the most dominant order was *Methanosarcinales* (ranging from 50 to 57%) followed by *Methanobacteriales* (in the range of 21–26%) in all reactors. The archaeal community structure on the genus level is shown in Figure 7.

In all samples, *Methanosarcina* was the most dominant genus (from 43 to 51% of relative abundance). These archaea are often abundant in the reactors during anaerobic digestion of nitrogen-rich manure [21,38] and generate CH_4_ using each of the known methanogenic pathways [41]. The relative abundance of the strong acetoclastic genus *Methanothrix* was lower than that of the genus *Methanosarcina* in all treatments, considering the relatively higher tolerance of members of the genus *Methanosarcina* to high levels of organic acids and ammonium [41]. Moreover, some species of the genera *Methanosarcina* and *Methanothrix* can also participate in the DIET mechanism [42,43]. Representatives of the genera *Methanobacterium* and *Methanoculleus* were also found in all reactors, indicating that they are the key participants in hydrogenotrophic methanogenesis under various conditions. Other studies have also identified these genera as widespread methanogens in different anaerobic biogas-producing systems [21,44].

**Figure 8:**
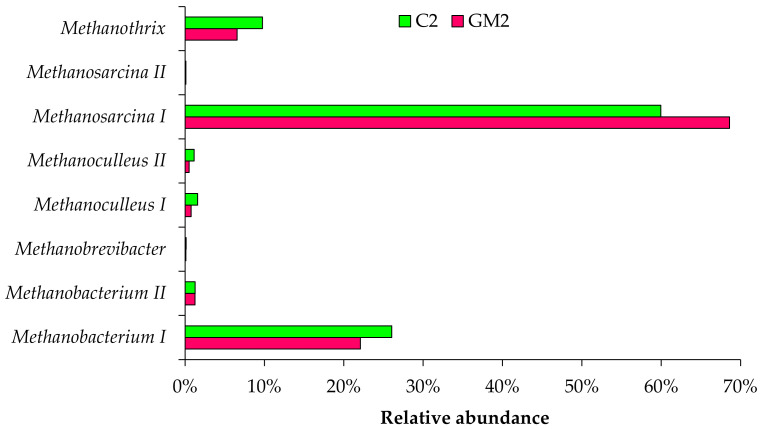
Taxonomic composition of methanogenic communities in the anaerobic reactors according to amplicon sequencing of *mcrA* gene (sampled on day 6).

In the case of the control reactor and the reactor GM2, methanogenic consortia were additionally investigated by the *mcrA* gene amplicon high-throughput sequencing approach (Figure 8). Thus, more than 60 thousand high-quality *mcrA* gene sequences were obtained for both samples. The *mcrA* gene approach allowed a higher level of *Methanosarcina* and a lower level of *Methanothrix* and *Methanobacterium* in the reactor with the addition of GAC and magnetite to be detected. The relative abundance data received by using the *mcrA* gene give better results, since data obtained with the use of the 16S rRNA gene approach are more biased because of the different copy numbers of rRNA operons in different archaeal taxa, while *mcrA* is a single-copy gene in most methanogenic archaea.

There is now an increasing focus on adding carbonaceous materials, zerovalent iron, and iron oxide minerals such as magnetite to anaerobic systems because these additives can increase CH_4_ production without any modification to existing anaerobic systems. It was noted that the enhancement of methanogenesis is mediated, among other factors, by the enhancement of direct interspecies electron transfer between representatives of anaerobic microbial communities [45,46,47,48]. However, the response to the addition of additives is not universal, as the negative impact of the addition of individual additives on microbial communities and the anaerobic process has been reported [29,49]. Thus, the effect of supplements considered in the context of DIET on the structure and activity of anaerobic communities is still under investigation. It has recently been demonstrated that the composition of microbial communities, and hence the metabolic pathways during anaerobic digestion, differ depending on different types of conductive materials and the type of substrate used, as well as various process parameters [20,21,46].

## 4. Conclusions

In summary, our results show that the addition of magnetite nanopowder, GAC, and biochar into biogas reactors has a positive effect on the process of anaerobic digestion of chicken manure. Although the addition of magnetite, GAC, and biochar improved methanogenic performance, the reactors with the combined addition of GAC and magnetite functioned better than other systems. Thus, the maximum methane production rate from these reactors increased significantly up to 48% compared to the control reactors. Taxonomic profiles indicate that both bacterial and archaeal communities involved in the anaerobic conversion of chicken manure in the absence/presence of various additives did not change significantly among the samples (based on the 16S rRNA gene analysis). Various bacterial groups within the classes *Bacteroidia* and *Clostridia* dominated in the reactors. Representatives of the genera *Methanosarcina*, *Methanobacterium*, *Methanothrix*, and *Methanoculleus* were mostly observed within the archaeal communities in the reactors. On the contrary, the *mcrA* gene approach allowed a higher level of *Methanosarcina* in the system with GAC/Fe_3_O_4_ to be detected. However, the mechanism underlying the effects of accelerating agents on the methane yield potential from these substrates requires further study.

## Figures and Tables

**Table 1:** Alpha diversity of microbial communities in the anaerobic reactors (sampled on day 6).

Reactor	Bacteria				Archaea			
	OTUs	Chao1	Shannon	Simpson	OTUs	Chao1	Shannon	Simpson
C2	454	464	6.20	0.94	48	49	3.88	0.88
M2	470	488	6.34	0.96	49	49	3.92	0.88
G2	468	471	6.28	0.95	49	49	3.99	0.89
GM2	463	466	6.30	0.95	49	49	3.90	0.88
B2	472	475	6.60	0.97	49	50	4.07	0.90
BM2	472	484	6.68	0.97	48	49	3.69	0.86

## Data Availability

The data presented in this study are available on request from the corresponding author.

## References

[1] Li K., Liu R., Sun C. (2015). Comparison of anaerobic digestion characteristics and kinetics of four livestock manures with different substrate concentrations. Bioresour. Technol..

[2] Tonanzi B., Crognale S., Gianico A., Della Sala S., Miana P., Zaccone M.C., Rossetti S. (2021). Microbial community successional changes in a full-scale mesophilic anaerobic digester from the start-up to the steady-state conditions. Microorganisms.

[3] Maurya R., Tirkey S.R., Rajapitamahuni S., Ghosh A., Mishra S., Hosseini M. (2019). Recent advances and future prospective of biogas production. Advances in Feedstock Conversion Technologies for Alternative Fuels and Bioproducts.

[4] Srivastava R.K. (2019). Bio-energy production by contribution of effective and suitable microbial system. Mater. Sci. Energy Technol..

[5] Yan C., Zhu L., Wang Y. (2016). Photosynthetic CO_2_ uptake by microalgae for biogas upgrading and simultaneously biogas slurry decontamination by using of microalgae photobioreactor under various light wavelengths, light intensities, and photoperiods. Appl. Energy.

[6] Ziganshina E.E., Bulynina S.S., Ziganshin A.M. (2021). Assessment of *Chlorella sorokiniana* growth in anaerobic digester effluent. Plants.

[7] Ziganshina E.E., Bulynina S.S., Ziganshin A.M. (2022). Growth characteristics of *Chlorella sorokiniana* in a photobioreactor during the utilization of different forms of nitrogen at various temperatures. Plants.

[8] Niu Q., Takemura Y., Kubota K., Li Y.Y. (2015). Comparing mesophilic and thermophilic anaerobic digestion of chicken manure: Microbial community dynamics and process resilience. Waste Manag..

[9] Nie H., Jacobi H.F., Strach K., Xu C., Zhou H., Liebetrau J. (2015). Monofermentation of chicken manure: Ammonia inhibition and recirculation of the digestate. Bioresour. Technol..

[10] Nahm K.H. (2005). Factors influencing nitrogen mineralization during poultry litter composting and calculations for available nitrogen. Worlds Poult. Sci. J..

[11] Westerholm M., Moestedt J., Schnurer A. (2016). Biogas production through syntrophic acetate oxidation and deliberate operating strategies for improved digester performance. Appl. Energy.

[12] Yekta S.S., Svensson B.H., Björn A., Skyllberg U. (2014). Thermodynamic modeling of iron and trace metal solubility and speciation under sulfidic and ferruginous conditions in full scale continuous stirred tank biogas reactors. Appl. Geochem..

[13] Antoni D., Zverlov V.V., Schwarz W.H. (2007). Biofuels from microbes. Appl. Microbiol. Biotechnol..

[14] Zhao Z., Zhang Y., Li Y., Dang Y., Zhu T., Quan X. (2017). Potentially shifting from interspecies hydrogen transfer to direct interspecies electron transfer for syntrophic metabolism to resist acidic impact with conductive carbon cloth. Chem. Eng. J..

[15] Ziganshina E.E., Belostotskiy D.E., Bulynina S.S., Ziganshin A.M. (2021). Effect of magnetite on anaerobic digestion of distillers grains and beet pulp: Operation of reactors and microbial community dynamics. J. Biosci. Bioeng..

[16] Yang Y., Zhang Y., Li Z., Zhao Z., Quan X., Zhao Z. (2017). Adding granular activated carbon into anaerobic sludge digestion to promote methane production and sludge decomposition. J. Clean. Prod..

[17] Wang T., Zhang D., Dai L., Dong B., Dai X. (2018). Magnetite triggering enhanced direct interspecies electron transfer: A scavenger for the blockage of electron transfer in anaerobic digestion of high-solids sewage sludge. Environ. Sci. Technol..

[18] Capson-Tojo G., Moscoviz R., Ruiz D., Santa-Catalina G., Trably E., Rouez M., Crest M., Steyer J.P., Bernet N., Delgenes J.P. (2018). Addition of granular activated carbon and trace elements to favor volatile fatty acid consumption during anaerobic digestion of food waste. Bioresour. Technol..

[19] Pan J., Ma J., Liu X., Zhai L., Ouyang X., Liu H. (2019). Effects of different types of biochar on the anaerobic digestion of chicken manure. Bioresour. Technol..

[20] Ziganshina E.E., Belostotskiy D.E., Bulynina S.S., Ziganshin A.M. (2020). Influence of granular activated carbon on anaerobic co-digestion of sugar beet pulp and distillers grains with solubles. Processes.

[21] Ziganshina E.E., Bulynina S.S., Ziganshin A.M. (2022). Impact of granular activated carbon on anaerobic process and microbial community structure during mesophilic and thermophilic anaerobic digestion of chicken manure. Sustainability.

[22] Ziganshina E.E., Sagitov I.I., Akhmetova R.F., Saleeva G.T., Kiassov A.P., Gogoleva N.E., Shagimardanova E.I., Ziganshin A.M. (2020). Comparison of the microbiota and inorganic anion content in the saliva of patients with gastroesophageal reflux disease and gastroesophageal reflux disease-free individuals. BioMed Res. Int..

[23] Jang H.M., Choi Y.K., Kan E. (2018). Effects of dairy manure-derived biochar on psychrophilic, mesophilic and thermophilic anaerobic digestions of dairy manure. Bioresour. Technol..

[24] Jang H.M., Brady J., Kan E. (2021). Succession of microbial community in anaerobic digestion of dairy manure induced by manure-derived biochar. Environ. Eng. Res..

[25] Gerardi M.H. (2003). The Microbiology of Anaerobic Digesters.

[26] Fagbohungbe M.O., Herbert B.M.J., Hurst L., Ibeto C.N., Li H., Usmani S.Q., Semple K.T. (2017). The challenges of anaerobic digestion and the role of biochar in optimizing anaerobic digestion. Waste Manag..

[27] Suanon F., Sun Q., Mama D., Li J., Dimon B., Yu C.P. (2016). Effect of nanoscale zero-valent iron and magnetite (Fe_3_O_4_) on the fate of metals during anaerobic digestion of sludge. Water Res..

[28] Ziganshin A., Ziganshina E., Byrne J., Gerlach R., Struve E., Biktagirov T., Rodionov A., Kappler A. (2015). Fe(III) mineral reduction followed by partial dissolution and reactive oxygen species generation during 2,4,6-trinitrotoluene transformation by the aerobic yeast *Yarrowia lipolytica*. AMB Express.

[29] Luo C., Lü F., Shao L., He P. (2015). Application of eco-compatible biochar in anaerobic digestion to relieve acid stress and promote the selective colonization of functional microbes. Water Res..

[30] Lovley D.R. (2017). Syntrophy goes electric: Direct interspecies electron transfer. Annu. Rev. Microbiol..

[31] Wei Y., Li Z., Ran W., Yuan H., Li X. (2021). Performance and microbial community dynamics in anaerobic co-digestion of chicken manure and corn stover with different modification methods and trace element supplementation strategy. Bioresour. Technol..

[32] Krieg N.R., Staley J.T., Brown D.R., Hedlund B.P., Paster B.J., Ward N.L., Ludwig W., Whitman W.B. (2010). Bergey’s Manual of Systematic Bacteriology.

[33] Detman A., Bucha M., Treu L., Chojnacka A., Pleśniak Ł., Salamon A., Łupikasza E., Gromadka R., Gawor J., Gromadka A. (2021). Evaluation of acidogenesis products’ effect on biogas production performed with metagenomics and isotopic approaches. Biotechnol Biofuels.

[34] Hahnke S., Langer T., Koeck D.E., Klocke M. (2016). Description of *Proteiniphilum saccharofermentans* sp. nov., *Petrimonas mucosa* sp. nov. and *Fermentimonas caenicola* gen. nov., sp. nov., isolated from mesophilic laboratory-scale biogas reactors, and emended description of the genus *Proteiniphilum*. Int. J. Syst. Evol. Microbiol..

[35] Chen S., Dong X. (2005). *Proteiniphilum acetatigenes* gen. nov., sp. nov., from a UASB reactor treating brewery wastewater. Int. J. Syst. Evol. Microbiol..

[36] Lesnik K.L., Cai W., Liu H. (2019). Microbial community predicts functional stability of microbial fuel cells. Environ. Sci. Technol..

[37] Li R., Duan N., Zhang Y., Liu Z., Li B., Zhang D., Dong T. (2017). Anaerobic co-digestion of chicken manure and microalgae *Chlorella* sp.: Methane potential, microbial diversity and synergistic impact evaluation. Waste Manag..

[38] Bi S., Westerholm M., Qiao W., Xiong L., Mahdy A., Yin D., Song Y., Dong R. (2020). Metabolic performance of anaerobic digestion of chicken manure under wet, high solid, and dry conditions. Bioresour. Technol..

[39] Evans P.N., Parks D.H., Chadwick G.L., Robbins S.J., Orphan V.J., Golding S.D., Tyson G.W. (2015). Methane metabolism in the archaeal phylum *Bathyarchaeota* revealed by genome-centric metagenomics. Science.

[40] Yu T., Wu W., Liang W., Lever M.A., Hinrichs K., Wang F. (2018). Growth of sedimentary *Bathyarchaeota* on lignin as an energy source. Proc. Natl. Acad. Sci. USA.

[41] De Vrieze J., Hennebel T., Boon N., Verstraete W. (2012). *Methanosarcina*: The rediscovered methanogen for heavy duty biomethanation. Bioresour. Technol..

[42] Rotaru A.E., Shrestha P.M., Liu F., Markovaite B., Chen S., Nevin K.P., Lovley D.R. (2014). Direct interspecies electron transfer between *Geobacter metallireducens* and *Methanosarcina barkeri*. Appl. Environ. Microbiol..

[43] Rotaru A.E., Shrestha P.M., Liu F., Shrestha M., Shrestha D., Embree M., Zengler K., Wardman K., Nevin K.P., Lovley D.R. (2014). A new model for electron flow during anaerobic digestion: Direct interspecies electron transfer to *Methanosaeta* for the reduction of carbon dioxide to methane. Energy Environ. Sci..

[44] Braga Nan L., Trably E., Santa-Catalina G., Bernet N., Delgenès J.P., Escudié R. (2020). Biomethanation processes: New insights on the effect of a high H_2_ partial pressure on microbial communities. Biotechnol. Biofuels.

[45] Lin R., Cheng J., Zhang J., Zhou J., Cen K., Murphy J.D. (2017). Boosting biomethane yield and production rate with graphene: The potential of direct interspecies electron transfer in anaerobic digestion. Bioresour. Technol..

[46] Lee S.H., Kang H.J., Lim T.G., Park H.D. (2020). Magnetite and granular activated carbon improve methanogenesis via different metabolic routes. Fuel.

[47] Zhao Z., Li Y., Zhang Y., Lovley D.R. (2020). Sparking anaerobic digestion: Promoting direct interspecies electron transfer to enhance methane production. iScience.

[48] Zhang J., Lu T., Zhong H., Shen P., Wei Y. (2021). Zero valent iron improved methane production and specifically reduced aminoglycoside and tetracycline resistance genes in anaerobic digestion. Waste Manag..

[49] Zhang J., Wang Z., Wang Y., Zhong H., Sui Q., Zhang C., Wei Y. (2017). Effects of graphene oxide on the performance, microbial community dynamics and antibiotic resistance genes reduction during anaerobic digestion of swine manure. Bioresour. Technol..

